# Catecholamine levels with use of electronic and combustible cigarettes

**DOI:** 10.18332/tid/190687

**Published:** 2024-08-14

**Authors:** Remy Poudel, Shen Li, Haoyun Hong, Juan Zhao, Shweta Srivastava, Rose Marie Robertson, Jennifer L. Hall, Sanjay Srivastava, Naomi M. Hamburg, Aruni Bhatnagar, Rachel J. Keith

**Affiliations:** 1 American Heart Association, Dallas, United States; 2 Department of Medicine, Envirome Institute, University of Louisville, Louisville, United States; 3 Whitaker Cardiovascular Institute, Chobanian & Avedisian School of Medicine, Boston University, Boston, United States

**Keywords:** electronic cigarettes, catecholamine, biomarker, cardiovascular risk

## Abstract

**INTRODUCTION:**

Smoking elevates catecholamines that increase the risk for cardiovascular disease. Sparse evidence exists about the effects of e-cigarettes and catecholamines. Higher levels of catecholamines could trigger the increased heart rate, blood pressure, and decreased vascular function reported with the use of e-cigarettes. We investigated the difference in urinary catecholamines and their metabolites before and after the use of an e-cigarette containing nicotine or cigarettes compared to no tobacco use.

**METHODS:**

In our observational cohort exposure study, healthy adults aged 21–45 years who were currently using e-cigarettes, cigarettes, or had never used tobacco, participated in an acute exposure visit using their most common tobacco product. Urine was collected before, 1, and 2 hours after a 3-second puff every 30 seconds for 10 minutes on an e-cigarette or straw or use of 1 cigarette. Urinary catecholamines and their metabolites were measured by ultra-high-performance liquid chromatography. Participants (n=323) were grouped by the product used at the visit. We compared levels of creatinine normalized log-transformed urinary catecholamines and their metabolites across groups using Dunn’s test following a Kruskal-Wallis test in unadjusted and demographically adjusted models.

**RESULTS:**

Prior to use, individuals who used cigarettes (n=70) had lower urinary metabolites from epinephrine, serotonin, and norepinephrine. No differences were seen in those who used e-cigarettes (n=171) and those who did not use tobacco (n=82). In fully adjusted models, 1 h after the use of a combustible or e-cigarette, log-transformed urinary metabolites from norepinephrine (β=1.22; 95% CI: 0.39–2.05, p=0.004 and β=1.06; 95% CI: 0.39–1.74, p=0.002), dopamine (β=0.37; 95% CI: 0.24–0.5, p<0.001 and β=0.15; 95% CI: 0.05–0.26, p<0.001), and epinephrine (β=1.89; 95% CI: 0.51–3.27, p=0.008 and β=1.49; 95% CI: 0.38–2.61, p=0.009) were elevated. In fully adjusted models, combustible cigarette use was associated with elevated urinary norepinephrine (β=0.46; 95% CI: 0.13–0.81, p=0.007) and dopamine (β=0.19; 95% CI: 0.06–0.31, p=0.003) 1 h after use.

**CONCLUSIONS:**

We found that the use of both e-cigarettes and cigarettes was associated with elevated urinary catecholamines or their metabolites. Catecholamines could be useful as a biomarker of harm for tobacco use and considered by tobacco regulatory scientists in future research.

## INTRODUCTION

Smoking and other forms of tobacco or nicotine use, including the use of e-cigarettes, induce biomarkers of cardiovascular disease (CVD) risk^1^. Smoking combustible cigarettes and exposure to nicotine stimulates catecholamine release^2^ and sympathetic tone. Smoking-related catecholamine stimulation is associated with increased heart rate (HR), blood pressure (BP), and coronary vasoconstriction, which are risk factors for CVD^3^. The tobacco-induced increase in catecholamines may be partly responsible for the increased CVD risk associated with cigarette use and has also been linked to more serious cardiovascular complications^4^. Recent work suggests e-cigarette use, including pod-based devices, alters HR, BP, and arterial health measured as flow-mediated dilation (FMD)^5^. An increase in sympathetic tone may cause these changes. There is a gap in the literature about the relationship between the use of e-cigarettes and catecholamine levels or whether changes in catecholamine levels may mediate the heart rate and vascular effects seen with the use of these products.

Increased sympathetic tone occurs when the ‘flight or fight’ response is triggered, in part, by catecholamine release and leads to increased heart rate, constricted blood vessels, and increased blood pressure. Chronically increased stress responses, including chronic increases in catecholamines, can contribute to cardiovascular disease^6^. This stress response, and indirectly sympathetic tone, can be assessed by investigating the catecholamines that help regulate the sympathetic nervous system activity (i.e. norepinephrine, dopamine)^7^ and adrenal medullary secretion (i.e. epinephrine)^8^. Specifically, high blood pressure has been linked to elevated epinephrine and dopamine metabolite 3-methoxytyramine^8^, while platelet aggregation and secretion have been linked to epinephrine and norepinephrine^9^. High levels of catecholamines damage myocardial cells and contribute to cardiac vascular remodeling^10^. Chronically increased levels of catecholamines may contribute to chronic heart failure^11^ and arrhythmia^12^. Therefore, catecholamines are a relevant and important biomarker of harm that is associated with combustible cigarettes, but has not been rigorously explored with e-cigarette use.

Many users of e-cigarettes initiate use due to the reduced harm claims. Despite these early claims, questions have arisen about their safety, with evidence of potential changes to cardiovascular function associated with e-cigarette use, some of which could be related to changes in sympathetic tone and catecholamine levels. This study aims to identify changes in the urinary levels of catecholamines and their metabolites with acute usage of e-cigarettes and combustible cigarettes or no tobacco use. These changes are indicative of cardiovascular risk and may provide more information on the harm reduction claims associated with e-cigarettes. It can also help build a library of biomarkers of interest for regulating tobacco products.

## METHODS

### Study design

To evaluate tobacco product use and cardiovascular risk, participants were recruited into the investigator-initiated Cardiovascular Injury due to Tobacco Use (CITU) 2.0 study from July 2018 to July 2020, as previously described^13^. This study is an observational cohort study to explore longitudinal change in risk biomarkers during an acute usage study visit repeated one year apart. Study visits were scheduled after an 8-h food fast and a 6-h tobacco fast. All study visits occurred before 11 a.m. to limit the effects of circadian changes. Each visit included a structured interview on demographics, socioeconomics, lifestyle, health, family history of heart disease, allergies, and tobacco use. A detailed self-reported history of tobacco use was collected using a modified version of the National Health Interview Survey on tobacco use, and surveys were harmonized with the PhenX toolkit to include detailed information on e-cigarette and non-traditional tobacco products. The in-person study visit started with baseline urine collection (T0) and pregnancy screening. The participants then proceeded to our specialized exposure room and remained supine for a 10-minute rest period. After the rest period, all vascular and heart rate variability measures were completed, and a blood draw was performed. Participants were then positioned in a seated fashion to complete their structured exposure session. Immediately following the exposure session, participants were placed in a supine position again. Repeated vascular measures and heart rate variability were completed. The participants then provided a 1-h post-exposure urine sample (T1). A second blood draw, lung function testing, anthropometric measures, and questionnaires were completed in the next hour. The third urine sample was collected 2 hours after the exposure session (T2) completion. A centralized laboratory at the UoL processed and performed urinary and blood measurements. All surveys were collected and kept in Research Electronic Data Capture (REDCap), a secure web application for building and managing online surveys and databases^14^. Each institutional review board approved CITU 2.0 and all participants provided written consent (BU #H-32613 and UofL #18.1259).

### Participants

Participants were recruited from two sites (Boston, MA, and Louisville, KY) and included self-reported healthy participants aged 21–45 years who were non-tobacco users (<100-lifetime uses), e-cigarette users (>20-lifetime vape sessions and current use for the past six months for at least three days per week) or current smokers (>100-lifetime cigarettes and current use for the past three months at least three days per week).

### Exclusion criteria

**Figure 1:**
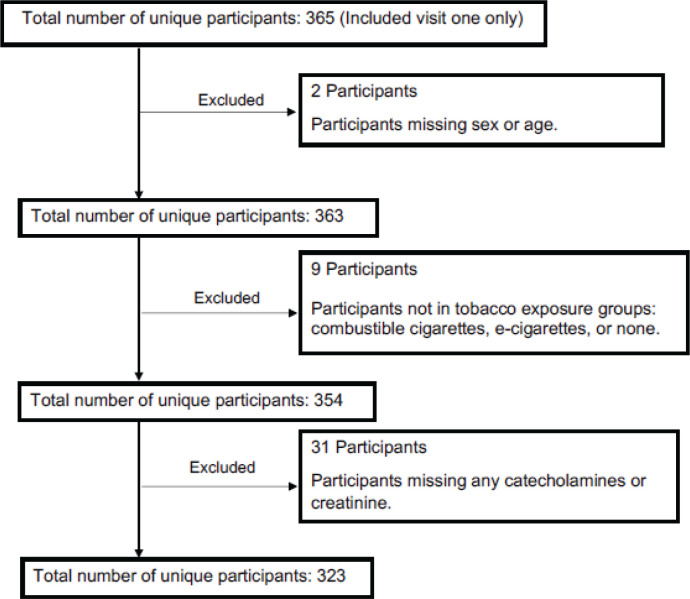
Consort flow diagram of participant inclusion in the Cardiovascular Injury due to Tobacco Use from 2018–2020, Boston and Louisville, United States (N=323)

At the time of this study, there were 365 participants in the CITU 2.0 cohort. Criteria for exclusion included missing demographic data needed for adjustment (n=2), use of cigarillos on the day of their visit, or use of a product that may have contained cannabis instead of a nicotine vape liquid (n=9). Individuals without urinary measures of catecholamines at baseline, at 1 h post-exposure, and at 2 h post-exposure, were also excluded from the study (n=31). The final sample size consisted of 323 participants (Figure 1).

### Study protocol

*Acute tobacco exposure session*

After completion of the baseline measures, participants were asked to complete a structured use protocol for smoking, vaping, or sham (those who do not use tobacco products) exposure within our specially designed exposure study rooms. Participants were asked to bring their typical product for this session, with dual users bringing their most used e-cigarette. Those who vaped were asked to bring a new vape or filled device of their most commonly used brand, flavor, and nicotine concentration. They then used their device for one three-second puff every 30 seconds for ten minutes. If participants reported they could not handle the nicotine content from two puffs per minute, they could reduce their puffs per minute. Those who smoked were asked to use a whole combustible cigarette in no more than 10 minutes. Those who had never used tobacco products were asked to inhale on a straw for three seconds every 30 seconds for ten minutes. Urine and vascular measures were collected as described, before the session and at one and two hours after product use.

*Catecholamine measurements*

As described before^15^, UPLC-MS/MS was used to analyze urinary levels of free biogenic monoamines (including catecholamines), and their metabolites were measured by 5 µL of urine samples from frozen biobanked (-80°C) random samples were thawed on ice and mixed with 50 µL of deuterated internal standards (epinephrine-d6, norepinephrine-d6, dopamine-d4, metanephrine-d3, normetanephrine-d3, 4-hydroxy-3-methoxymandelic acid d3, 4-hydroxy-3-methoxyphenyl-d3-acetic-d2 acid, 5-hydroxyindole-4,6,7-d3-3-acetic-d2 acid) and 195 µL of 0.2% formic acid in a 2 mL amber UPLC sample vial and the samples were analyzed on a Xevo TQ-S micro quadrupole mass spectrometer with an ESI ionization source, interfaced with Waters Acquity Class-H UPLC equipped with a quaternary pump system (Waters, MA). The analytes were resolved on an Acquity UPLC high strength silica (HSS) perfluorophenyl (PFP) (150 mm × 2.1 mm, 1.8 µm) column (Waters Inc., MA) maintained at 40°C, using a binary gradient consisting of 0.2% formic acid (Solvent A) and methanol (Solvent B). The gradient started with 0.5% solvent B at a flow rate of 0.4 mL/min for 1 min and then ramped up to 95% solvent B at a flow rate of 0.35 mL/min over a period of 3 min. The gradient was then maintained at these conditions for 0.5 min before recycling back to 0.5% solvent B in 0.1 min and then held at 0.5% solvent B at a flow rate of 0.4 mL/min for 5.4 min. The MS/MS data were acquired in time-scheduled multiple reaction ion monitoring (MRM) mode using electrospray ionization. Polarity switching detected positive and negative ions in the same run. The electrospray ionization inlet conditions were capillary 0.50 kV, cone 28 V, source temperature 150°C, desolvation temperature 600°C, cone gas flow 50 L/h, and desolvation gas flow 1000 L/h. Supplementary file Figure 1 shows the catecholamines and metabolites measured in the present study.

### Statistical analysis

Participants’ baseline characteristics were tabulated and stratified into three categories, including those who used combustible e-cigarettes, cigarettes, or no tobacco, based on participants’ product use during the study visit. Descriptive statistics are represented as percentages for categorical variables, and means with standard deviation or medians with range for continuous variables. Differences in descriptive statistics between the non-tobacco user group and two tobacco use groups (combustible cigarette use and e-cigarette use) were assessed using the Kruskal-Wallis test for continuous variables and Pearson chi-squared tests for categorical variables. After normalizing our urinary metabolites to creatinine, the data were not normally distributed, so the data were natural-log-transformed, making them more symmetric and easier to analyze^16^.

The study outcomes were catecholamines and metabolites, normalized by urine creatinine, where cotinine is divided by creatinine and then multiplied by 100. Generalized linear models were then natural-log-transformed. We conducted modeled outcomes that led to the extraction of crude β coefficients (Model 1) and adjusted β coefficients (Model 2, adjusting for age, sex, race, ethnicity, normalized cotinine, and site), which were used to estimate the associations between the outcomes and tobacco product use across the time points. For each outcome, at each time point, two models were fitted: 1) Model 1 (unadjusted); and 2) Model 2 (age, sex, race, ethnicity, normalized cotinine, and site (Louisville University vs Boston University). The β coefficients in our regression models indicated the change in outcomes (catecholamines and metabolites that were normalized by urine creatinine then log-transformed) associated with being in either the combustible smokers or e-cigarette smokers’ groups, compared to the reference group (non-smokers), while controlling for other variables included in the model. Statistical significance was assessed at α<0.05. Additional sensitivity analysis was performed to explore and assess the influence of outcome outliers. Specifically, the upper 5% of each catecholamine or metabolite was systematically removed, and the subsequent influence on the analysis was assessed. We conducted a power calculation based on the current sample size of the three groups. With an effect size of 0.5, an alpha level of 0.05, and a two-tailed significance test, we have a power exceeding 80%. Data were analyzed on the American Heart Association Precision Medicine Platform using the open-source software R (v4.2.0, R Foundation for 219 Statistical Computing, Vienna, Austria).

## RESULTS

As seen in Table 1, the mean age of the participants was 25.8 ± 6.8 years; 48% reported their biological sex as male, 37.2% self-reported being part of a minority race, and 10.8% reported coming from a Hispanic, Latino, or Spanish ethnic group. Participants who smoked were significantly older (aged 31 years), more likely to report their race as Black or African American, and were less likely to report belonging to a Hispanic, Latino, or Spanish ethnic group (all p<0.05). Participants who used an e-cigarette were significantly aged younger at 24 years than those who did not use tobacco products (aged 26 years) (p<0.05). Participants who smoked exhibited higher heart rate, systolic blood pressure, and diastolic blood pressure at baseline, as well as higher levels of creatinine (all p<0.05). Both those who smoked and vaped had higher residual levels of baseline cotinine, a nicotine metabolite, at baseline (p<0.05).

**Table 1:** Baseline characteristics of healthy adults aged 21–45 years from the Cardiovascular Injury due to Tobacco Use 2.0 observational cohort study population recruited at UL and BU from 2018–2020 categorized by product used during the acute exposure study visit, Boston and Louisville, United States (N=323)

*Characteristics*	*No tobacco use (N=82) n (%)*	*E-cigarette use (N=171) n (%)*	*Cigarette use (N=70) n (%)*	*Total (N=323) n (%)*
**Age** (years), mean ± SD	25.8 ± 5.98	**23.7 ± 5.84**	**31.1 ± 7.19**	25.8 ± 6.83
**Race**				
Asian	20 (24.4)	42 (24.6)	15 (21.4)	77 (23.8)
Black/African American	1 (1.2)	11 (6.4)	**8 (11.4)**	20 (6.2)
Other/AIAN	6 (7.3)	12 (7.0)	4 (5.7)	22 (6.8)
White	55 (67.1)	105 (61.4)	43 (61.4)	203 (62.8)
Don’t know	0 (0)	1 (0.6)	0 (0)	1 (0.3)
**Ethnicity**				
Hispanic, Latino or Spanish	9 (11.0)	25 (14.6)	**1 (1.4)**	35 (10.8)
**Sex**				
Female	34 (41.5)	90 (52.6)	**43 (61.4)**	167 (51.7)
Male	48 (58.5)	81 (47.4)	**26 (37.1)**	155 (48.0)
Intersex	0 (0)	0 (0)	1 (1.4)	1 (0.3)
**Heart rate** (beats/min), mean ± SD	62.4 ± 9.31	63.2 ± 9.38	**67.9 ± 10.8**	64.0 ± 9.89
**SBP** (mmHg), mean ± SD	111 ± 10.8	114 ± 11.6	**116 ± 13.4**	114 ± 11.9
**DBP** (mmHg), mean ± SD	68.4 ± 8.18	70.4 ± 7.80	**73.7 ± 9.83**	70.6 ± 8.55
**Creatinine** (mg/dL), mean ± SD	128 ± 101	158 ± 102	**172 ± 115**	153 ± 106
**Normalized cotinine,** mean ± SD	3.77 ± 3.65	**674 ± 741**	**855 ± 912**	543 ± 757

Continuous variables are assessed using the Kruskal-Wallis test (followed by Dunn test). Categorical variables are assessed using Pearson’s chi-squared test. Bold text indicates statistical differences between no tobacco as the reference, and tobacco product use groups. A p<0.05 indicates statistically significant findings.

**Figure 2:**
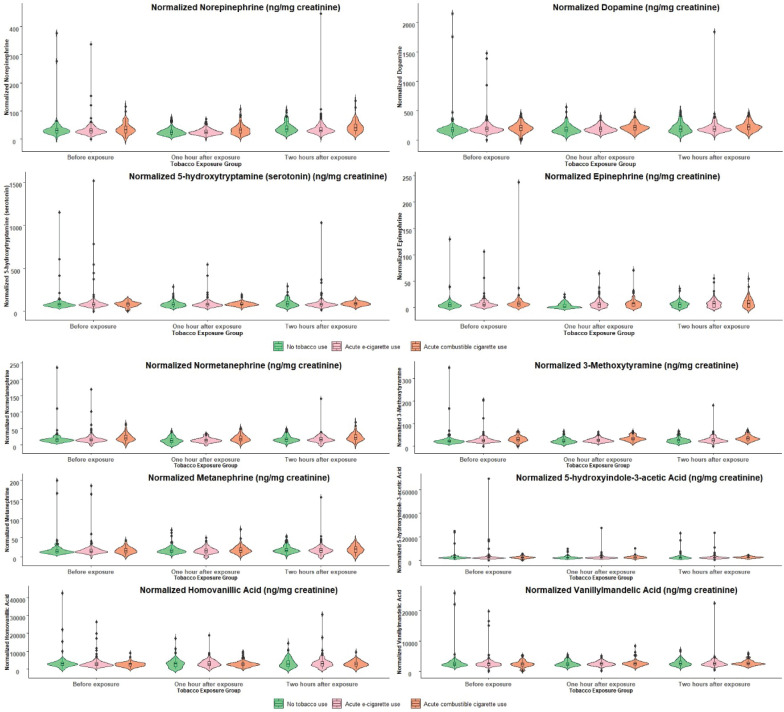
Levels of urinary catecholamines and their metabolites normalized to creatinine (ng/mg creatinine) from healthy adults aged 21–45 years in Cardiovascular Injury due to Tobacco Use 2.0 observational cohort study population recruited at UL and BU from 2018–2020 categorized by products used during acute exposure study visit before (T0), and at 1 h (T1) and 2 h (T2) after exposure, Boston and Louisville, United States (N=323)

Levels of log-transformed catecholamines and their metabolites were normalized to creatinine and compared between the individuals after either no tobacco use, e-cigarette use, or combustible cigarette use at baseline, at 1h, and 2 h after the exposure session (Figure 2). A sensitivity analysis was performed to remove the upper 5% of the variables and assess the influence of outliers. There were no differences, so the full dataset was used in the modeling analysis.

Catecholamines and their metabolites were normalized to creatinine and then log-transformed for general linear analysis in unadjusted and adjusted models with a priori selected demographic variables that may confound catecholamine or creatinine levels (Tables 2–4). Table 2 compares all pre-exposure samples (T0) across product use. Compared to those who did not use any tobacco products, only combustible users had differences in catecholamine metabolites with lower metanephrine (β= -0.45; 95% CI: -0.88– -0.02, p=0.04), 5-hydroxyindole-3-acetic acid (β = -0.65; 95% CI: -1.18– -0.12, p=0.02) and vanillylmandelic acid (β= -0.7; 95% CI: -1.14 – -0.25, p=0.002) for fully adjusted models.

**Table 2:** Generalized linear analysis of urinary levels of log-transformed normalized catecholamines and their metabolites prior to a tobacco exposure session (N=323)

*Catecholamines and metabolites*	*Categories*	*Model 1*	*Model 2*
		*β (95% CI)*	*β (95% CI)*
Norepinephrine	Combustible cigarette	-0.01 (-0.41–0.39)	-0.33 (-0.78–0.12)
	E-cigarette	-0.08 (-0.42–0.25)	-0.14 (-0.51–0.23)
Dopamine	Combustible cigarette	-0.11 (-0.51–0.3)	-0.38 (-0.83–0.07)
	E-cigarette	-0.07 (-0.40–0.26)	-0.13 (-0.49–0.24)
5-hydroxytryptamine (serotonin)	Combustible cigarette	-0.10 (-0.43–0.22)	-0.27 (-0.63–0.10)
	E-cigarette	-0.12 (-0.39–0.15)	-0.18 (-0.48–0.12)
Normetanephrine	Combustible cigarette	0.1 (-0.36–0.55)	-0.20 (-0.72–0.31)
	E-cigarette	-0.26 (-0.63–0.12)	-0.33 (-0.75–0.09)
3-Methoxytyramine	Combustible cigarette	0.06 (-0.29–0.40)	-0.2 (-0.59–0.19)
	E-cigarette user	-0.001 (-0.29–0.29)	-0.03 (-0.35–0.28)
Metanephrine	Combustible cigarette	-0.18 (-0.56–0.2)	**-0.45 (-0.88 – -0.02)**
	E-cigarette	-0.17 (-0.49–0.14)	-0.27 (-0.62–0.08)
Epinephrine	Combustible cigarette	**1.11 (0.12–2.09)**	0.83 (-0.29–1.96)
	E-cigarette	0.8 (-0.06–1.58)	0.81 (-0.11–1.72)
5-hydroxyindole-3-acetic acid	Combustible cigarette	-0.28 (-0.76–0.2)	**-0.65 (-1.18 – -0.12)**
	E-cigarette	-0.22 (-0.61–0.17)	-0.26 (-0.69–0.18)
	E-cigarette	0.42 (-0.17–1.01)	0.31 (-0.35–0.97)
	E-cigarette	-0.16 (-0.5–0.17)	-0.17 (-0.53–0.2)

Model 1: unadjusted. Model 2: adjusted for age, sex, race, ethnicity, normalized cotinine, and site. Bold text indicates statistical differences between sham session as the reference group (no tobacco) and tobacco product use groups. A p<0.05 indicates statistically significant findings.

**Table 3:** Generalized linear analysis of urinary levels of log-transformed normalized catecholamines and their metabolites 1 hour after a tobacco exposure session (N=323)

*Catecholamines and metabolites*	*Categories*	*Model 1*	*Model 2*
		*β (95% CI)*	*β (95% CI)*
Norepinephrine	Combustible cigarette	0.23 (-0.07–0.52)	**0.46 (0.12–0.81)**
	E-cigarette	-0.1 (-0.34–0.14)	0.16 (-0.13–0.43)
Dopamine	Combustible cigarette	**0.18 (0.07–0.29)**	**0.19 (0.06–0.31)**
	E-cigarette	0.06 (-0.04–0.15)	0.05 (-0.05–0.15)
5-hydroxytryptamine (serotonin)	Combustible cigarette	0.04 (-0.07–0.16)	0.06 (-0.07–0.19)
	E-cigarette	-0.01 (-0.10–0.09)	0.02 (-0.09–0.12)
Normetanephrine	Combustible cigarette	**1.43 (0.73–2.13)**	**1.22 (0.39–2.05)**
	E-cigarette	**0.96 (0.38–1.54)**	**1.06 (0.39–1.74)**
3-Methoxytyramine	Combustible cigarette	**0.37 (0.26–0.49)**	**0.37 (0.24– 0.5)**
	E-cigarette user	**0.13 (0.04–0.23)**	**0.15 (0.05–0.26)**
Metanephrine	Combustible cigarette	-0.12 (-0.72–0.48)	0.12 (-0.58–0.83)
	E-cigarette	**-0.62 (-1.11 – -0.12)**	-0.44 (-1.01–0.14)
Epinephrine	Combustible cigarette	**2.92 (1.6–4.25)**	**3.52 (1.99–5.03)**
	E-cigarette	**2.49 (1.39–3.59)**	**3.36 (2.12–4.6)**
5-hydroxyindole-3-acetic acid	Combustible cigarette	0.09 (-0.02–0.21)	0.08 (-0.05–0.21)
	E-cigarette	-0.04 (-0.14–0.05)	-0.03 (-0.14–0.08)
Homovanillic acid	Combustible cigarette	**1.68 (0.54–2.83)**	**1.89 (0.51–3.27)**
	E-cigarette	**1.33 (0.38–2.27)**	**1.49 (0.38–2.61)**
Vanillylmandelic acid	Combustible cigarette	0.03 (-0.08–0.15)	0.09 (-0.05–0.22)
	E-cigarette	-0.03 (-0.12–0.07)	0.04 (-0.07–0.15)

Model 1: unadjusted. Model 2: adjusted for age, sex, race, ethnicity, normalized cotinine, and site. Bold text indicates statistical differences between sham session as the reference group (no tobacco) and tobacco product use groups. A p<0.05 indicates statistically significant findings.

**Table 4:** Generalized linear analysis of urinary levels of log-transformed normalized catecholamines and their metabolites 2 hours after a tobacco exposure session (N=323)

*Catecholamines and metabolites*	*Categories*	*Model 1*	*Model 2*
		*β (95% CI)*	*β (95% CI)*
Norepinephrine	Combustible cigarette	0.11 (-0.13–0.35)	0.12 (-0.16–0.40)
	E-cigarette	-0.16 (-0.35–0.04)	-0.07 (-0.31–0.16)
Dopamine	Combustible cigarette	0.14 (0.02–0.27)	0.12 (-0.02–0.26)
	E-cigarette	0.02 (-0.08–0.13)	-0.03 (-0.14–0.09)
5-hydroxytryptamine (serotonin)	Combustible cigarette	0.01 (-0.12–0.13)	-0.03 (-0.18–0.12)
	E-cigarette	-0.04 (-0.15–0.07)	-0.09 (-0.21–0.04)
Normetanephrine	Combustible cigarette	0.15 (-0.46–0.75)	-0.28 (-0.1–0.43)
	E-cigarette	-0.36 (-0.87–0.14)	-0.41 (-1.00–0.19)
3-Methoxytyramine	Combustible cigarette	0.33 (0.11–0.55)	0.22 (-0.04–0.48)
	E-cigarette user	0.02 (-0.16–0.20)	-0.002 (-0.22–0.21)
Metanephrine	Combustible Cigarette	-0.51 (-1.22–0.20)	-0.27 (-1.14–0.6)
	E-cigarette	-0.54 (-1.13–0.05)	-0.23 (-0.95–0.5)
Epinephrine	Combustible cigarette	0.28 (-1.06–1.61)	0.38 (-1.19–1.95)
	E-cigarette	0.13 (-0.98–1.23)	0.77 (-0.55–2.08)
5-hydroxyindole-3-acetic acid	Combustible cigarette	0.02 (-0.11–0.14)	-0.04 (-0.19–0.11)
	E-cigarette	-0.1 (-0.21–0.01)	**-0.15 (-0.28 – -0.03)**
Homovanillic acid	Combustible cigarette	0.07 (-1.17–1.31)	1.37 (-0.12–2.85)
	E-cigarette	-0.59 (-1.62–0.43)	0.85 (-0.39–2.09)
Vanillylmandelic acid	Combustible cigarette	0.002 (-0.11–0.11)	0.02 (-0.11–0.15)
	E-cigarette	-0.04 (-0.13–0.05)	-0.01 (-0.11–0.10)

Model 1: unadjusted. Model 2: adjusted for age, sex, race, ethnicity, normalized cotinine, and site. Bold text indicates statistical differences between sham session as the reference group (no tobacco) and tobacco product use groups. A p<0.05 indicates statistically significant findings.

In Table 3, we compare urinary levels of catecholamines and their metabolites 1 h after exposure (T1) between those who used a combustible cigarette or an e-cigarette to those who completed a sham exposure session. One hour after exposure, in fully adjusted models, compared to individuals with a sham use session, those who used combustible cigarettes had higher levels of norepinephrine (β=0.46; 95% CI: 0.13–0.81, p=0.007), dopamine (β=0.19; 95% CI: 0.06–0.31, p=0.003), normetnephrine (β=1.22; 95% CI: 0.39–2.05, p=0.004), 3-methoxytyramine (β=0.37; 95% CI: 0.24–0.5, p<0.001), epinephrine (β=3.52; 95% CI: 1.99–5.03, p<0.001) and homovanillic acid (β=1.89; 95% CI: 0.51–3.27, p=0.008). When compared to individuals with a sham use session, individuals who used an e-cigarette, after fully adjusting, had a significant positive association with normetanephrine (β=1.06; 95% CI: 0.39–1.74, p=0.002), 3-methoxytyramine (β=0.15; 95% CI: 0.05–0.26, p=0.005), epinephrine (β=3.36; 95% CI: 2.12–4.6, p<0.001) and homovanillic acid (β=1.49; 95% CI: 0.38–2.61, p=0.009) at 1 h post-exposure (Table 3). In fully adjusted models, at 2 h after the use of an e-cigarette, urinary levels of 5-hydroxyindole-3-acetic acid were significantly lower than levels seen in individuals with sham use (β= -0.15; 95% CI: -0.28 – -0.03, p=0.02) (Table 4).

## DISCUSSION

In this study, we recruited healthy young adults who use e-cigarettes, combustible cigarettes, or never use tobacco products and had them undergo a structured use session. Those who used combustible cigarettes had lower baseline urinary levels of metabolites from epinephrine, serotonin, and norepinephrine, while those who used e-cigarettes had similar levels of urinary metabolites to those who did not use tobacco. One hour after use of an e-cigarette or cigarette, urinary levels of metabolites from norepinephrine, dopamine, and epinephrine were elevated. Combustible cigarette use also led to higher levels of urinary norepinephrine and dopamine one hour after use. By two hours after use, most urinary biomarkers were similar across use groups. This study demonstrates changes in urinary catecholamines and their metabolites after the use of e-cigarettes.

Though others have reported higher levels of catecholamines after smoking^17^, our findings suggest vaping may also cause repeat stimulation of the sympathetic nervous system. Catecholamine levels, including repeated elevations, have significant implications for cardiovascular health. Nicotine induces the adrenal medulla to release epinephrine, activating the sympathetic nervous system. Increased levels of epinephrine are associated with high blood pressure^8^, and repeated elevations can contribute to the risk of atherosclerosis and CVD^18^. Nicotine-induced catecholamine exposure is linked to arrhythmias, inappropriate shocks from cardiac defribillators^19^, and decreased heart rate variability. Catecholamine levels are associated with stress cardiomyopathy^20^, and the increased sympathetic tone seen with acute myocardial infarction^21^. Similar to epinephrine, dopamine is released in response to low levels of nicotine and may increase sympathetic tone, contributing to the health consequences and the addictive nature of tobacco^22^. If overstimulated chronically with the use of e-cigarettes, the sympathetic nervous and adrenal medullary systems could contribute to both the health consequences and the addictive nature of these products. Indeed, emerging studies have shown that the use of an e-cigarette containing nicotine increases heart rate and both systolic and diastolic blood pressure^23^. Other studies suggest that vascular tone and function are negatively impacted after the use of an e-cigarette^24^. Given the elevation in catecholamines reported here with e-cigarette use and the exposure to nicotine, our study elucidates one possible pathway for the physiological changes seen with vaping, namely activation of the sympathetic nervous system and adrenergic receptors.

Understanding the potential health and addiction implications of e-cigarettes has been limited by the continually changing nature of the products. Over the previous decade, electronic devices have transitioned from delivering low levels of nicotine to newer generations that have the potential to deliver nicotine and other potentially toxic compounds in doses similar to cigarettes^25^. Despite ongoing changes to the devices, research suggests that e-cigarettes have a unique nicotine delivery profile^26^. Given the differences seen in the delivery of nicotine, we may not be able to rely on interpretations of the data on catecholamine release from combustible cigarette use. Increases in catecholamine levels vary with different tobacco products, and these variations are hypothesized to relate to the amount of nicotine exposure^27^. Furthermore, the nicotine delivery route elicits varied physiological responses^28^. Not only does product type and route of nicotine delivery create a differential sympathetic response, but the duration of use also creates variations in catecholamine release. Chronic smokers have a catecholamine response to lower doses of nicotine, suggesting changes in nicotinic cholinergic receptors with chronic exposure^29^. Given the variations of physiological response based on nicotine exposure, catecholamine response to e-cigarette use is likely to be unique, making the relationship between e-cigarette use and catecholamine levels timely and of regulatory importance.

Nicotine is not the only potentially toxic exposure from tobacco products; there are >7000 toxins in combustible cigarettes, and at least ten have been identified in e-cigarettes. While there are still gaps in understanding how specific constituents of tobacco products contribute to toxicity, much of combustible cigarette toxicity may be attributed to the combustion products, which include large amounts of tobacco alkaloids, aldehydes, volatile organic compounds (VOC’s), and particulate matter (PM). Evidence suggests anabasine, a tobacco alkaloid produced with cigarette smoking, exerts agonistic activity on nicotinic receptors, contributing to the sympathetic effects of tobacco smoke^30^. Though cigarette use increases anabasine, the use of e-cigarettes has shown little to no appreciable levels^31^. Nevertheless, e-cigarettes do contain nicotine and other harmful or potentially harmful constituents (HPHCs), sometimes at levels similar to traditional cigarettes^25^. Thus, even though e-cigarettes have the potential to deliver high levels of nicotine, the catecholamine release may be affected by differences in the overall exposure profile of HPHCs among sole users of e-cigarettes compared to users of combustible cigarettes.

Our study presents differences in the catecholamine profile of those who used e-cigarettes and cigarettes since combustible cigarettes alone elevated levels of the parent compounds. Dopamine, epinephrine, norepinephrine, and serotonin all have plasma half-lives of around 2 minutes, strikingly shorter than the hour or more half-life of the metabolites. We chose to study the urinary metabolites of compounds instead of the parent compounds alone since they can provide unique exposure profiles over time. Still, metabolism can be affected by numerous different factors. Catecholamines are metabolized by several enzymes, including monoamine oxidases (MAO) and cytochrome P450 enzymes, including cytochrome P450 2A6 (CYP2A6), which modulates nicotine metabolism^32^. Demographic features, dietary substances, and smoking behaviors alter the activity of CYP2A6^33^, which can vary the metabolism of nicotine and catecholamines.

Furthermore, MAO is inhibited by combustible cigarettes^34^, which has important implications for catecholamine metabolism. This study cannot ascertain whether the differences in the parent catecholamines with the use of combustible cigarettes reflect a difference in nicotine exposure, pharmacokinetics, or metabolism. Thus, future research would be important to address this issue.

### Limitations

There are potential limitations to our study. We chose to use urinary catecholamines to optimize the potential to collect both parent catecholamines and metabolites based on the longer half-life of these compounds, but this measure is affected by urine dilution. To address this, we used urinary creatinine to normalize our data. We did not have an observed tobacco and nicotine fast. Instead, we excluded individuals with urinary cotinine >500 ng/mL at baseline. Postural changes between our urine collection time points could have led to decreased levels of catecholamines; we saw an increase in catecholamines and their metabolites, suggesting these postural changes were minimal compared to the effects of the tobacco products. Finally, we allowed participants to use their preferred tobacco product as part of a standard exposure protocol. We believe the inclusion of multiple products contributes to the generalizability of the study as the effects we present are across a range of combustible cigarettes and e-cigarette devices and flavors, strengthening the argument that the effect seen is related to a class of tobacco products, not just one device. There could also be residual confounding related to study design or participant demographics of which we are unaware.

## CONCLUSIONS

We found that both the use of e-cigarettes and cigarettes is associated with elevated urinary catecholamines and their metabolites, suggesting that the use of either product could increase CVD risk due to repeated sympathetic stimulation.

## Supplementary Material



## Data Availability

The data underlying this article will be shared on reasonable request to the corresponding author.
