# Supplementary material for: Catecholamine levels with use of electronic and combustible cigarettes
Source: Tob Induc Dis. 2024 Aug 14;22:10.18332/tid/190687. doi: 10.18332/tid/190687 (PMC11320712; doi:10.18332/tid/190687)
Supplement: Supplementary file 1 [file TID-22-144-s1.pdf]

Appendix 1. Phase I metabolites of catecholamines

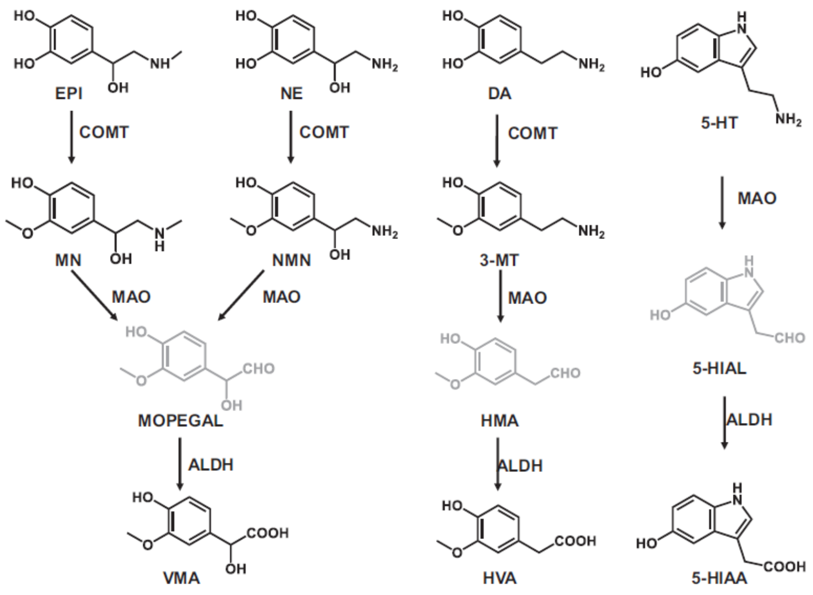

Scheme 1. Phase I metabolites of biogenic monoamines. Epinephrine (EPI), norepinephrine (NE), dopamine (DE), metanephrine (MN), normetanephrine (NMN) 3-methoxytyramine (3-MT), serotonin (5-HT), 5-hydroxyindole-3-acetic acid (5-HIAA), vanillylmandelic acid (VMA), homovanillic acid (HVA), 3-methoxy-4-hydroxyphenylglycolaldehyde (MOPEGAL), 4-hydroxy-3-methoxyphenylacetaldehyde (HMA) and 5-hydroxyindole-3-acetaldehyde (5-HIAL). The two enzymes are catechol-*O*-methyltransferase (COMT) and L-monoamine oxidase (MAO).

©2024 Poudel R. et al.
